# Alternative Promoters of *GRIK2* (*GluR6*) Gene in Human Carcinoma Cell Lines Are Regulated by Differential Methylation of CpG Dinucleotides

**DOI:** 10.3390/genes13030490

**Published:** 2022-03-10

**Authors:** Vikramjit K. Zhawar, Raj P. Kandpal, Raghbir S. Athwal

**Affiliations:** 1 Fels Institute for Cancer Research and Molecular Biology, Lewis Katz School of Medicine, Temple University, Philadelphia, PA 19067, USA; vikram97jit@pau.edu; 2 Department of Basic Medical Sciences, Western University of Health Sciences, Pomona, CA 91766, USA

**Keywords:** carcinoma, *GluR6*, *GRIK2*, *GluR6* variants, epigenetic regulation, neuronal and non-neuronal promoters in *GluR6*/*GRIK2*, CpG methylation in *GluR6* promoters, bisulfite sequencing, luciferase reporter

## Abstract

The ionotropic glutamate receptor 6 (*GluR6* or *GRIK2*) gene is transcribed by two cell-type-specific promoters in neuronal and non-neuronal cells, which results in five different transcript variants. The purpose of this study was to explore cell-type-specific silencing of these promoters by epigenetic mechanisms. The neuronal and non-neuronal promoter sequences were cloned upstream of the luciferase gene in the pGL3 luciferase reporter vector. Promoter susceptibility to methylation was confirmed by 5-azacytidine and trichostatin treatment, and the status of CpG dinucleotides was determined by bisulfite sequencing of the promoter was determined by bisulfite sequences. GluR6A transcript variant was expressed in the brain, and GluR6B was most abundant in tumor cell lines. The neuronal promoter was methylated in non-neuronal cell lines. The treatment with 5-azacytidine and trichostatin upregulated transcription of the *GluR6* gene, and methylation of the GluR6 promoter sequence in the luciferase reporter system led to downregulation of the luciferase gene transcription. Bisulfite sequencing revealed methylation of 3 and 41 CpG sites in non-neuronal and neuronal promoters, respectively. The differential activation/silencing of GluR6 promoters suggests that the transcript variants of GluR6 are involved in tissue-specific biological processes and their aberrant regulation in tumor cells may contribute to distinct properties of tumor cells.

## 1. Introduction

The ionotropic glutamate receptors are multimeric ligand-gated channels that regulate the flux of sodium and calcium ions across the neuronal cell membranes. These receptors are classified based on their binding preference for N-methyl-D-aspartate (NMDA), α-amino-3-hydroxy-5-methyl-4-isoazolepropionic acid (AMPA), and 2-carboxy-3-carboxymethyl-4-isopropenylpyrrolidine (kainate) [1]. The kainate class consists of GluR5, GluR6 (GRIK2), GluR7, KA1, and KA2. While KA1 and KA2 require heteromerization with GluR5/6/7 for functional channels, GluR5, GluR6, and GluR7 are capable of functioning both as homomers and heteromers [2,3]. GluR6, a member of the kainic acid subgroup of glutamate receptors, has been studied for neurotransmission and kainic acid-induced nerve cell death [4,5].

The *GluR6* (*GRIK2*) gene was first mapped to chromosome 6q [6], and subsequently localized within a 900 Kb region on 6q21 [7]. The loss of heterozygosity for markers D6S1543, D6S449, D6S283, and D6S434 in breast cancer, prostate cancer, pancreatic cancer, colorectal cancer, lymphomas, and leukemia are suggestive of the presence of a tumor critical gene in this region of chromosome 6 [8,9,10,11,12]. The relevance of GluR6 for tumorigenesis is further confirmed by alterations in its sequence due to 6q deletions observed in acute lymphocytic leukemia [13]. The biological significance of chromosome 6q is evident by its ability to induce senescence of normal and tumor cells [14,15,16]. By using the chromosome transfer technique, we have shown that the smallest region of chromosome 6 responsible for senescence harbors the *GluR6* gene. The gene is transcribed into five isoforms (A, B, C, D, and E) in a tissue-specific manner by alternative usage of two distinct promoters in neuronal and non-neuronal cells, respectively [7]. We have previously shown that the introduction of expression constructs of *GluR6* in normal fibroblasts and ovarian carcinoma cells induces senescence [17,18]. Our results indicate that GluR6 by itself is sufficient to induce senescence in tumor cells. The role of GluR6 in apoptosis is indirectly supported by other observations in the literature [5].

To address tissue-specific transcriptional regulation of GluR6 isoforms, we profiled the expression of the *GluR6* gene in a variety of tumor cell lines and investigated epigenetic regulation and transcriptional silencing of this gene in a cell-type-specific manner. Our results demonstrate transcriptional silencing or downregulation of *GluR6* gene transcription in a tissue-specific manner and implicate alternative functions of GluR6 in tumorigenesis.

## 2. Materials and Methods

### 2.1. Cell Culture

We used normal human fibroblast cells (FS2) as a control for transcript comparison with five ovarian carcinoma cell lines, four breast carcinoma cell lines, three melanoma cell lines, two glioblastoma cell lines, and one cell line each for prostate carcinoma, lung carcinoma, hepatoma, bladder carcinoma, and chemically immortalized fibroblasts. All cell lines (FS2, OVCAR429, OVCAR3, SKOV3, PA-1, HEY8A, SKBR-3, MCF-7, T47D, MDAMB468, WM239A, WM266A, WM983B, T98G, U87MG, DU145, A549, FOCUS, EJ, and SUSM1) were acquired from American Type Culture Collection, Manassas, VA, USA. The cells were grown in DMEM-F12 medium (VWR, Radnor, PA, USA) supplemented with 10% FBS and 10 mg/mL of streptomycin-penicillin (VWR). Cells were passaged at 85% confluence one day before harvesting the cultures for the isolation and purification of RNA and DNA. FS2 and SUSM1 represent normal and chemically transformed fibroblasts, respectively. Among the five ovarian carcinoma cell lines, three are adenocarcinomas (OVCAR429, SKOV3, OVCAR3), one advanced adenocarcinoma (HEY-A8), and one is a teratocarcinoma (PA-1); breast cancer cells are adenocarcinoma (SKBR3, MCF7, MDAMB468) and ductal carcinoma (T47D); melanoma cell lines (WM239A, WM266A, WM983B) are all metastatic; brain carcinoma cell lines (T98G, U87MG) have slightly differing stages of glioblastoma; prostate (DU145) and lung (A549) cell lines are both metastatic; FOCUS (hepatocellular carcinoma) and EJ (bladder carcinoma) have malignant potential. These carcinoma cell lines do not have significantly different metastatic/invasive characteristics. The main distinction is in the tissue of origin.

### 2.2. RNA Extraction, Reverse Transcription and Polymerase Chain Reaction (RT-PCR)

Total RNA was isolated from normal fibroblasts and tumor cells (10^6^–10^7^) by using Trizol (Thermo Fisher, Waltham, MA, USA) following the manufacturer’s protocol. The quality of RNA was assessed by measuring absorbance at 260 nm and 280 nm using a UV-spectrophotometer. Reverse transcription-polymerase chain reaction (RT-PCR) was used to amplify specific transcripts as below. Total RNA (0.5–2 μg) was mixed with 50–100 pmol oligodT and denatured at 72 °C for 5 min and cooled to 37 °C. The template RNA was transcribed into cDNA by adding 500 μM of each dNTP, 10 mM dithiothreitol, 1 U/mL of RNasin, and 200 U of Moloney murine leukemia virus (MMLV) reverse transcriptase (Thermo Fisher) in 1X transcription buffer to the RNA-oligodT heteroduplex in a volume of 20 μL and incubating the reaction mixture for 1 h at 37 °C. An aliquot of the cDNA was used for PCR as below.

For PCR, 25 pmol forward and reverse primers were mixed with 1 ng of cDNA, 200 mM each dNTP, 1.5 mM MgCl_2_, and 2.5 U of Taq polymerase (Promega, Madison, WI, USA) in 1X buffer (Promega) in a volume of 20 μL. Glyceraldehyde 3-phosphate dehydrogenase (GAPDH) primers were used to amplify the GAPDH transcript. The abundance of GAPDH transcripts was indicative of comparable amounts of cDNA used for amplifying *GluR6* transcripts. The primer sequences have been described previously [7]. PCR was carried out in a Perkin-Elmer thermocycler for 35 cycles consisting of 30 s/94 °C, 40 s/55 °C, and 1 min/72 °C.

### 2.3. DNA Extraction

Approximately 10^5^–10^6^ normal fibroblast or tumor cells were lysed in digestion buffer (100 mM NaCl, 10 mM Tris HCl of pH 8.0, 25 mM EDTA of pH 8.0, and 0.5% SDS) containing proteinase K (100 μg/mL) and incubated overnight at 55 °C. The cell lysates were extracted twice with a mixture of phenol: chloroform: isoamyl alcohol (25:24:1). The genomic DNA in the aqueous layer was precipitated with cold ethanol and spooled on a glass rod. The spooled DNA was dissolved in Tris-EDTA buffer (pH 8.0) containing 10 mM Tris-HCl and 1mM EDTA.

### 2.4. Southern Hybridization

Southern hybridization was carried out to investigate chromosomal rearrangements at the *GluR6* locus in tumor cells, and to analyze the methylation of MspI/HpaII restriction sites in *GluR6* promoters as follows. Several probe sequences corresponding to each 10–12 Kb region of the 900 Kb genomic stretch encompassing the *GluR6* gene were amplified by PCR using a series of primers. Restriction endonucleases were chosen to release 5–6 Kb fragments from the larger region of the genome, which were then probed by Southern hybridization to determine rearrangement of the genome around selected restriction sites. The methylation status of the *GluR6* promoter was analyzed by digesting genomic DNA with HpaII/MspI and probing the genomic DNA blots with promoter-specific fragments.

Briefly, DNA (10–20 mg) from tumor cell lines or normal cells was digested with an appropriate restriction endonuclease at 37 °C and electrophoresed on 0.8% agarose gel for 12–14 h at 30 volts and transferred to Hybond-XL (Amersham Pharmacia Biotech, Piscataway, NJ, USA) membrane. The membrane was hybridized to a P^32^-labeled DNA probe. The DNA fragments hybridizing to probe sequences were visualized by exposure to X-ray film.

### 2.5. Treatment with 5-Azacytidine and Trichostatin (TSA)

Selected tumor cell lines were grown in the medium described above with 0 to 5 mM 5-azacytidine (Sigma-Aldrich, St. Louis, MO, USA) for 6-days. The cells were harvested, and total RNA was isolated with Trizol (Thermo Fisher). In a different set of experiments, the above tumor cell lines were treated separately with 0.3 to 1 mM TSA (Sigma) for 36 h, and cells were harvested for RNA extraction. The quality of RNA was assessed by measuring absorbance in a UV spectrophotometer. RNA (0.5–2 μg) isolated from both sets of experiments were separately converted into cDNA in a volume of 20 μL, and a diluted aliquot of 1 ng cDNA was amplified by using Taq DNA polymerase in a Perkin-Elmer thermocycler to determine changes in the expression of GluR6 in treated cells as compared to untreated controls.

### 2.6. Bisulfite Sequencing

The methylation status of CpG dinucleotides in *GluR6* promoters of normal fibroblasts and tumor cell lines was determined by bisulfite sequencing as described [19]. Briefly, 1–2 μg of DNA was digested with the HindIII restriction enzyme and the digest was denatured in 0.3 N sodium hydroxide for 15 min at 37 °C. Unmethylated cytosines were sulphonated by incubation in 3.1 M sodium bisulfite (Sigma) and 0.5 mM hydroquinone (Sigma) at 55 °C for 16 h. The sulphonated DNA was purified using the Wizard DNA clean-up system (Promega) as per the manufacturer’s instructions. The purified DNA was incubated for 15 min in 0.3 N NaOH at 37 °C for de-sulphonation, precipitated with ethanol, and dissolved in distilled water for PCR.

The fragments specific for neuronal promoter (P_N_) and non-neuronal promoter (P_NN_) were amplified by PCR to include 90 CpG and 41 CpG dinucleotides of P_NN_ and P_N_ promoters, respectively [7]. PCR was performed in 50 μL reaction volume containing 5 μL of converted DNA, 200 mM each dNTP, 25 pmol of each primer, 2 mM MgCl_2_, and 2.5 U of Taq polymerase in 1X PCR buffer as described previously [7]. PCR conditions included denaturation at 95 °C for 10 min, followed by 30–40 cycles of 94 °C for 1 min, 50–55 °C for 1 min and 72 °C for 2 min and final elongation at 72 °C for 10 min. The amplified products were cloned in the pGEMT-easy vector system (Promega) by TA cloning. Four to six clones were sequenced to generate two distinct patterns of methylation corresponding to different chromosomes in the cells.

### 2.7. In Vitro Methylation of Promoter Constructs in Reporter Plasmid

The GC-rich regions of P_NN_ and P_N_ promoters were amplified from human DNA using sense primer P1F1 (with KpnI site; 5′TGCCTCGAGGGCCCATTAGG 3′; located at –7500 to −7487 to A of ATG on genomic DNA sequence) and 5′phosphorylated antisense primer P1R1 (5′AAGACTCGCAGTGAATCCGT3′; located at −5253 to −5274 relative to A of ATG) for P_NN_; sense primer P2F1 (with KpnI; 5′CAAACTGACTTTGATTTAGCTAG3′; located at –710 to –688 to A of ATG) and 5′phosphoylated antisense primer P2R1 (5′AGAGCGAACCGCCTGTTTA3′ located at +170 to +188) for P_N_. PCR was performed in 1 mM MgSO4, 300 mM of each dNTP, 50 pmol of each primer, and 2.5 U of Platinum Pfx DNA polymerase (Thermo Fisher) in 1X Pfx amplification buffer. The amplification was carried out by denaturation at 94 °C for 2 min followed by 35 cycles comprising denaturation at 94 °C for 15 s, annealing at 55 °C for 30 s, elongation at 68 °C for 3 min. PCR products were digested with KpnI and cloned in KpnI and SmaI sites of pGL3 Basic (Promega), sequenced, and named as −7500pGL3 (P_NN_) and −710-pGL3 (P_N_). Both constructs were either in vitro methylated by bacterial CpG methylase (SssI, from New England Biolabs, Ipswich, MA, USA) or mock treated in the absence of the enzyme. The methylation reaction was carried out by incubating DNA, SssI (1 U/mg of DNA), and S-adenosylmethionine in 1 × SssI buffer at 37 °C for 24 h. The DNA was purified with Qiagen spin columns and transfected into COS7 cells, U87MG cells, and SKOV3 cells along with the pRL-CMV vector (0.2 μg) as an internal control. After 48 h of transfection, cells were lysed and assayed for firefly and renilla luciferase using the Dual-Luciferase Reporter Assay System (Promega). The ratio of firefly to renilla luciferase activity was taken as a measure of relative luciferase activity (RLA). RLA of in vitro methylated construct was compared with the mock-treated construct.

## 3. Results

### 3.1. Expression Pattern of GluR6 in Tumor Cell Lines

**Figure 1:**
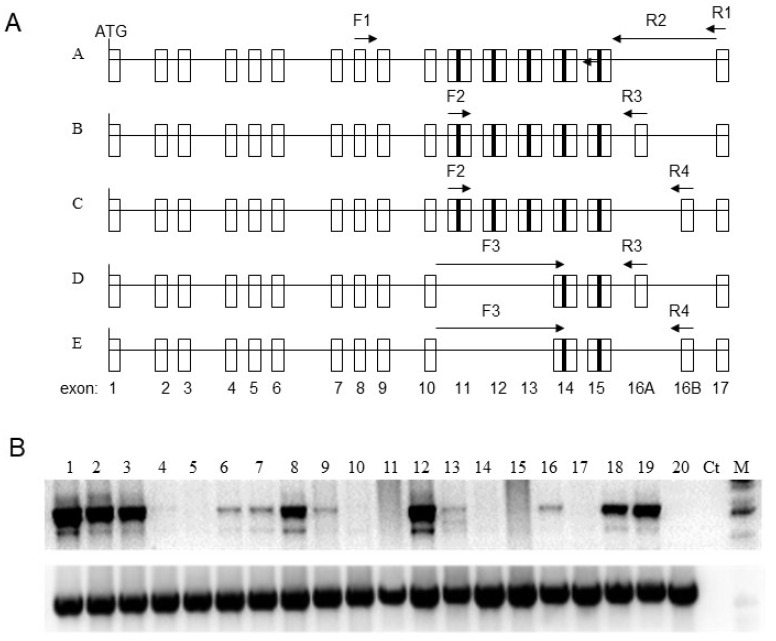
Expression of GluR6 isoforms in cell lines. (**A**) Positions of primers used for amplifying GluR6 alternative transcripts (A–E). The numbered open rectangles represent exons 1–17, and solid rectangle indicates the transmembrane domain. F1-R1 primers are common to all isoforms. F1-R2, F2-R3, F2-R4, F3-R3, and F3-R4 amplify GluR6 isoforms A, B, C, D, and E, respectively. (**B**) RNA responding to GluR6 was amplified with primers F1-R1 that are common to all isoforms. The bottom row shows relative levels of GAPDH transcript. Lane1: HDFs; lanes 2 and 3: WM239A and WM266A (melanoma); lane 4: A549 (lung); Lane 5: DU145 (prostate); lane 6: SUSM1 (chemically immortalized); lane 7: EJ (bladder); Lane 8: FOCUS (hepatoma); lane 9: T98G (glioblastoma); lanes 10 to 13: SKBR3, T47D, MDAMB468, and MCF7 (breast); Lanes 14 to 17: OVCAR429, PA1, SKOV3, and HEY8A (ovarian); lane 18: U87MG (glioblastoma); Lane 19: WM983B (melanoma) and lane 20: OVCAR3 (ovarian).

We investigated the expression of *GluR6* in 18 cell lines representing different tumors and compared them to the abundance of *GluR6* transcript in chemically immortalized fibroblasts (SUSM1) as well as normal fibroblasts (FS2). It has been previously reported that FS2 cells have a robust expression of four *GluR6* variants. To profile these variants, the cDNA was amplified by using primers specific to isoforms A, B, C, D, and E, respectively (Figure 1A).

The *GluR6* transcript was abundantly expressed in cell lines WM239A, WM266A, FOCUS, MDAMB468, U87MG, and WM983B (lanes 2, 3, 8, 12, 18, and 19 in Figure 1B). These cell lines correspond to melanoma, hepatoma, breast carcinoma, and glioblastoma. However, the expression was either very low or undetectable in other tumor cell lines used in this study. Although low levels of *GluR6* transcript were observed in SKOV3 (lane 16, Figure 1B), it was undetectable in other ovarian carcinoma cell lines. Similarly, MCF7 cells express low levels of *GluR6* transcript, but it was not detected in T47D and SKBR3 cells. These data do not allow a correlation between biological phenotypes and the abundance of *GluR6* transcripts.

As depicted in Figure 1A, specific combinations of primers can allow amplification of isoforms GluR6A (primers F2 and R2), GluR6B (primers F2 and R3), GluR6C (primers F2 and R4), GluR6D (primers F3 and R2), and GluR6E (primers F3 and R3). We used cDNA corresponding to fibroblast cells and human brain cDNA as controls. The normal fibroblasts showed expression of isoforms B, C, D, and E. However, brain cDNA was amplifiable with primers corresponding to isoform A only. Most tumor cell lines expressed the B isoform, the levels of which varied from modest to robust. As compared to the expression of GluR6B in tumor cell lines, GluR6D isoform was expressed in fewer cell lines. The isoforms C and E were present at very low levels in only a few cell lines.

### 3.2. Rearrangements of GluR6 Gene Locus in Tumor Cells

**Figure 2:**
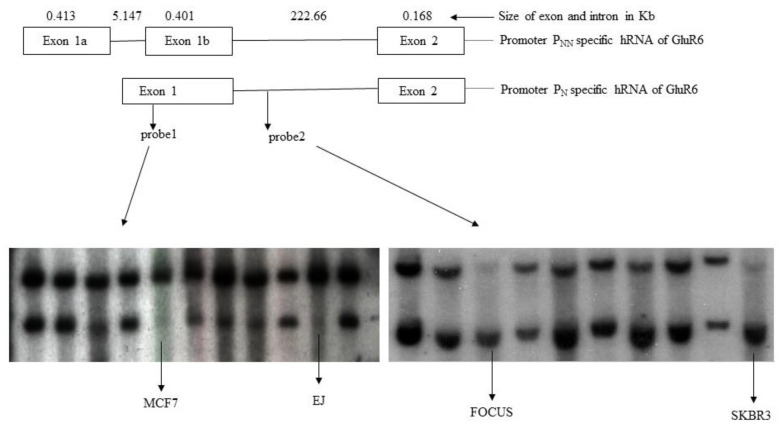
Southern hybridization of restriction enzyme digested DNA from tumor cell lines. Top two rows show the location of introns and exons transcribed by non-neuronal and neuronal promoters. The probes (probe 1 and probe 2) used for hybridization are indicated in the second row. The lanes showing rearrangements at *GluR6* locus in MCF7, FOCUS, EJ, and SKBR3 cells are marked.

To address the loss of expression or aberrant expression of *GluR6* isoforms in tumor cell lines, we have considered the possibility of tumor-specific genomic rearrangements. This was tested by generating unique PCR amplified probes from 10 Kb intervals of the 900 Kb genomic region of the *GluR6* locus. The genomic structure consisting of exons 1, 1a, 1b, and 2 of the gene and the locations of two probes used for demonstrating possible rearrangement are shown. The DNA was digested and probed with specific fragments. Any deviation from the expected pattern was considered a rearrangement (Figure 2).

We analyzed all cell lines but only a subset of tumor cell lines relative to FS2 fibroblasts are shown in Figure 2. The probes, designated as probe 1 and probe 2, originated from exon 1 and the intervening sequence between exon 1b and exon 2, respectively. As seen in Figure 2, most cell lines displayed the expected pattern of hybridization with probe 1, which was identical to the pattern observed for normal fibroblasts. Similar results were noticed for probe 2. While MCF7 and EJ genomic arrangement deviated from the expected pattern when analyzed with probe 1, genomic aberrations in SKBR3 and FOCUS cells are evident from the pattern of genomic DNA bands hybridizing to probe 2. These results suggest that genomic rearrangement might be responsible for aberrant expression or non-functional GluR6 in these cell lines.

### 3.3. Identification of CpG Rich Regions in Promoters of GluR6

We investigated epigenetic regulation of *GluR6* expression by characterizing previously described two distinct GluR6 promoters P_N_ and P_NN_ (7). The P_NN_ promoter is located approximately 5 kb upstream of promoter P_N_. P_NN_ is functional in fibroblast cells, and it has strong activity in both nerve cells (U87MG) as well as non-nerve cells (SKOV3). However, promoter P_N_ had fewer CpG dinucleotides and showed weaker activity as compared to P_NN_ both in nerve and non-nerve cells. P_N_ was the only promoter active in the human brain. The analysis of P_NN_ revealed it to have 71% CG bases with CpG islands located at −6313 to −6119 and −5887 to −5669, respectively (7). These CpG sites may account for differential transcriptional activation as described below.

### 3.4. Methylation Status of CpG Dinucleotides in Non-Neuronal Promoter (P_NN_) and Neuronal Promoter (P_N_)

**Figure 3:**
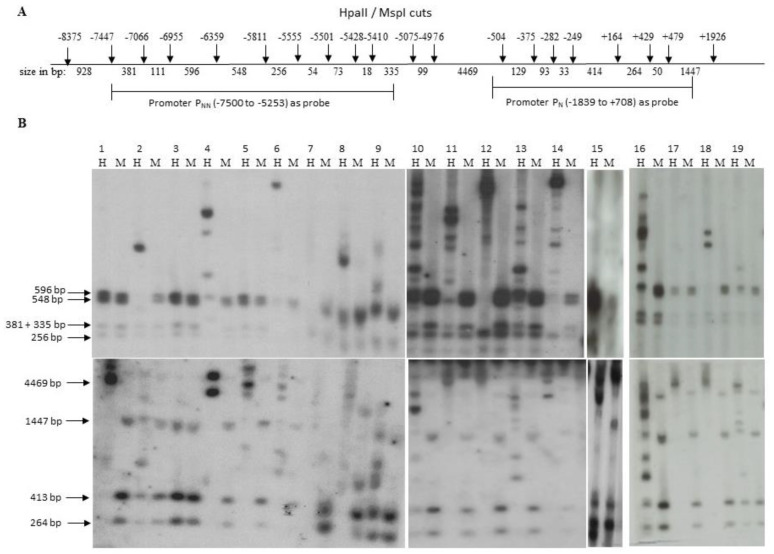
Identification of CpG methylation at HpaII/Msp I sites in promoter P_NN_ and promoter P_N_. (**A**) The locations of HpaII/MspI restriction sites relative to A of ATG on genomic DNA and restriction fragment lengths in base pairs for promoter regions corresponding to P_NN_ and P_N_ are indicated. (**B**) Southern blots of DNA of tumor cell lines and normal HDFs digested with HpaII (H) and MspI (M) and hybridized with P_NN_ specific probe (−7500 to −5253) and P_N_ specific probe (−1839 to +708) as shown in upper and lower panels, respectively. Lanes 1 to 19 correspond to the following cell lines. Normal HDFs; T47D; SkBr3; DU145; SKOV3; OVCAR429; PA1; OVCAR3; T98G; EJ; MCF7; FOCUS; OVCAR443; HEY8A; U87MG; MDAMB468; WM239A; WM983B; WM266A.

Figure 3A shows the locations of HpaII/MspI sites in P_N_ and P_NN_ promoters. These promoters were analyzed by Southern hybridization of HpaII/MspI digested DNA isolated from tumor cell lines. The blots of the digested DNA were hybridized to ^32^P-labeled fragments from promoter P_NN_ (−7500 to –5253) or promoter P_N_ (−1839 to +708). Restriction enzymes HpaII and MspI recognize the same nucleotide sequence (5′CCGG 3′), but they are differentially sensitive to the methylation of the CpG sequence at the restriction site. While MspI digestion is not affected by methylation, HpaII activity is methylation sensitive. As shown in Figure 3B, the P_N_ promoter was methylated in 18 of the 19 cell lines tested. However, methylation of the P_NN_ promoter was variable. The non-neuronal promoter P_NN_ was not methylated at any of the HpaII sites in normal cells and tumor cell lines WM239A, WM266A, and U87MG. However, P_N_ was methylated in both normal cells (FS2) as well as tumor cells. It is noteworthy that both promoters were not methylated in SKBR3. Although the SKBR3 promoters are not methylated, the absence of transcript may be explained by our unpublished observations suggesting deletion of the *GluR6* structural region in these cells.

### 3.5. Induction of GluR6 Expression by 5-Azacytidine (5-Aza) and Trichostatin (TSA)

**Figure 4:**
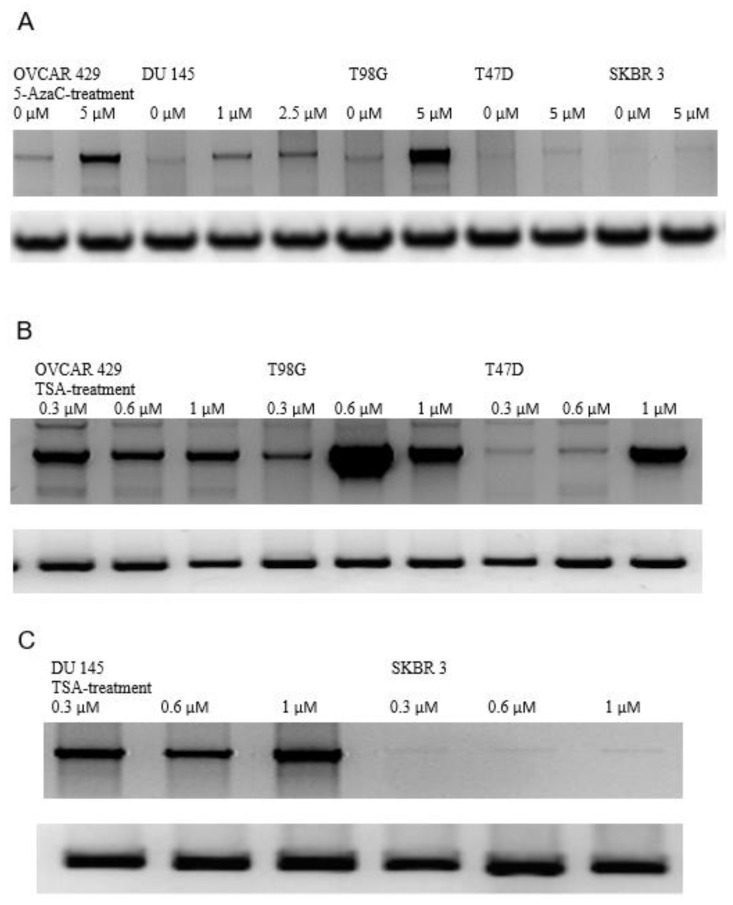
GluR6 expression in the presence of 5-azacytidine and TSA. (**A**) The cell lines were treated with 5-azacytidine, and the GluR6 transcript was amplified by RT-PCR. The cell lines and the treatment are indicated. (**B**,**C**) The cells were treated with TSA and the transcripts amplified by RT-PCR. The cell lines and the treatment are indicated. Amplification of GAPDH transcript to indicate comparable amounts of cDNA used for amplifying the transcript corresponding to GluR6 are shown in the bottom strip of each panel. The top portion of the panel shows amplification of GluR6 transcript. The amplified transcripts corresponding to GluR6 and GAPDH were electrophoresed in separate gels.

We selected five tumor cell lines that do not express *GluR6* transcripts. These cell lines included T47D, SKBR3, OVCAR429, DU145, and T98G. The cell lines were grown in the presence of 5-Aza (5–10 mM) for 6 days and analyzed for *GluR6* expression by RT-PCR using primers F1 and R1 (Figure 1). These primers are common for all GluR6 isoforms. As shown in Figure 4A, *GluR6* expression was induced in OVCAR429, DU145, and T98G in cells treated with 5-Aza. However, *GluR6* was not responsive to 5-Aza treatment in T47D and SKBR3. In a separate set of experiments, the cell lines were treated with TSA (0.3–1.0 mg/mL) for 36 h. It is evident from Figure 4B that *GluR6* transcription was induced in all tumor cell lines except SKBR3. The optimal concentration and length of treatment for 5- Azacytidine and TSA were determined prior to final experiments. The SKBR3 results are explainable by the deletion of the *GluR6* structural region as described in the previous section. These observations clearly demonstrate methylation-dependent silencing of GluR6 transcription in selected cell lines. Furthermore, transcriptional activation was also dependent on the acetylation of chromatin histones in these cells.

### 3.6. Inactivation of Promoter P_NN_ (−7500pGL3) and Promoter P_N_ (−710pGL3) by CpG Methylation

**Figure 5:**
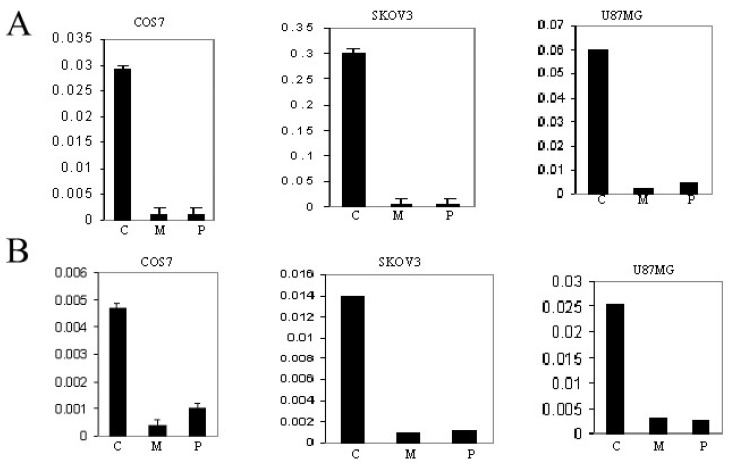
The effect of promoter methylation on the expression of luciferase reporter gene. (**A**) P_NN_ promoter containing construct was transfected into SKOV3, U87MG, and COS7 cells before and after methylation. The reporter gene expression was quantified by measuring the luminescence. (**B**) P_N_ promoter containing construct was transfected into SKOV3, U87MG, and COS7 cells before and after methylation. The reporter gene expression was quantified by measuring the luminescence, and the data represent mean + standard deviation of triplicate measurements. The *Y*-axis indicates relative luciferase activity (RLA) units. C, mock treated promoter; M, methylated promoter; P, empty pGL3 vector.

We selected the CpG-rich fragments that function as promoters (P_N_ and P_NN_) for cloning in the pGL3 vector as described previously [7]. These constructs are designated as −7500pGL3 (P_NN_) and −710pGL3 (P_N_). The constructs contain all CpG sequences of the two promoters. The activation of promoters was confirmed by measuring the luciferase activity. The effects of the constructs that were mock treated in CpG methylase buffer without SssI are designated as ‘C’ in the figure. The effects of the constructs methylated by SssI are designated as ‘M’. An empty vector designated as ‘P’ was used as a control. The constructs were transfected into U87MG, SKOV3, and COS7. As shown in Figure 5, the methylation of CpG dinucleotides in both promoters led to transcriptional inactivation of the luciferase gene in all three cell lines. These results confirm that the selected fragments function as promoters, and the activity of these promoters is inhibited by the methylation of CpG dinucleotides.

### 3.7. Bisulfite Sequencing of GluR6 Promoters in Tumor Cells

**Figure 6:**
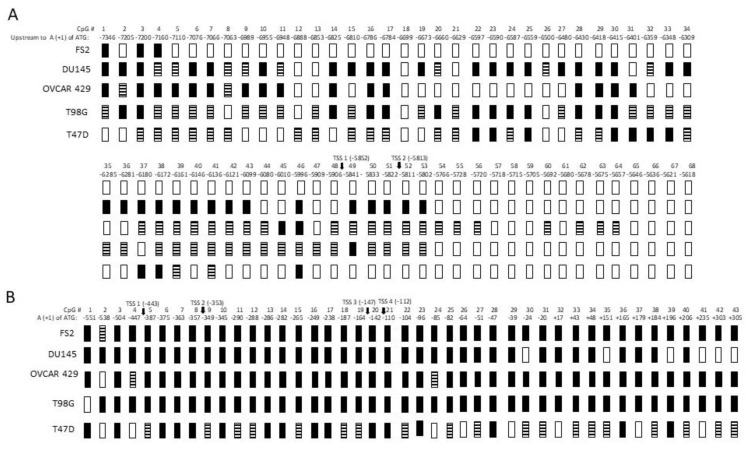
Methylation map of promoters P_NN_ panel (**A**) and P_N_ panel (**B**) in normal fibroblast cells (FS2) and four cancer cell lines (DU145, OVCAR429, T98G, T47D). Transcription start sites are indicated. Open rectangles denote unmethylated CpG, solid rectangles denote completely methylated CpG, and striped rectangles indicate methylated and some unmethylated clones. CpG sites 69–90 of P_NN_ promoter are not shown as all of them were unmethylated.

The methylation status of CpG sequences of the two promoters was analyzed in OVCAR429, T47D, DU145, T98G, and FS2 cell lines. Genomic DNA isolated from these cells was incubated with bisulfite to convert unmethylated cytosine residues to thymine. The converted DNA was amplified and cloned as described in the Methods section. The sequences of five clones for each amplified fragment were determined. The methylation of specific CpG sequences is presented in Figure 6.

A total of 87 CpG sites were unmethylated in the P_NN_ promoter of FS2 cells. In fact, sites 1, 3, and 4 were the only sites of this promoter that were methylated. All 41 CpG sites of the P_N_ promoter, however, were methylated. No methylation was observed for sites 67–90 of the P_NN_ promoter in all cell lines used. Of the remaining 66 sites, 44 sites in DU145, 42 sites in OVCAR429, 47 sites in T98G, and 28 sites in T47D were methylated. The P_N_ promoter in these cells was extensively methylated. The methylation of 36 sites in DU145, 40 sites in OVCAR429, 40 sites in T98G, and 31 sites in T47D was detected. The methylation of CpG sites in the promoter sequences explains the transcriptional silencing of GluR6 isoforms in neuronal and non-neuronal cells.

## 4. Discussion

Chromosome transfer technique has revealed the presence of a senescence causing region SEN6A on chromosome 6q21 [15,16]. In fact, the transfer of this region of the chromosome to a variety of carcinoma cells led to a senescence phenotype. The *GluR6* gene maps to the minimal region responsible for inducing senescence in tumor cells [17,18]. Although GluR6 has primarily been investigated in the context of excitable cells, unique isoforms of this gene have now been described in non-neuronal cells as well. We have previously described two promoters, P_N_ and P_NN_, that regulate the transcription of *GluR6* gene in neuronal and non-neuronal cells, respectively. While the neuronal promoter transcribes the *GluR6*A transcript isoform, the non-neuronal promoter activates the transcription of isoforms B, C, D, and E [7].

The promoter-specific expression of *GluR6* isoforms in neuronal and non-neuronal cells was confirmed by performing RT-PCR of mRNAs isolated from brain tissue, normal fibroblasts, and 18 tumor cell lines representing breast carcinoma, prostate carcinoma, ovarian carcinoma, melanoma, astrocytoma, and glioblastoma. The epigenetic regulation of promoters was substantiated by growing cells in the presence of 5-azacytidine and bisulfite sequencing of both promoters in cell lines. Methylation-dependent inhibition of transcription was demonstrated by using a luciferase reporter gene driven by the *GluR6* promoter.

The salient features of *GluR6* expression include the transcription of *GluR6B* isoform in most tumor cell lines. The expression of C, D, and E isoforms was also observed in some of these cell lines. Although *GluR6B* appears to be the dominant transcript, other isoforms are also present in these cell lines in detectable amounts. The quantitative analysis of transcripts does not appear to indicate a relationship between the biological properties of the cell lines and the transcript abundance. Southern hybridization of DNA from these cell lines did not show any conclusive evidence of chromosomal rearrangement. The possibility of such rearrangements and consequent aberrations in transcript sequence, however, cannot be ruled out.

The expression of *GluR6A* isoforms in neuronal cells and transcription of other isoforms in non-neuronal cells can be explained by cell line-specific methylation of two promoters. The results clearly demonstrate that the P_N_ promoter was methylated at nearly all HpaII sites in normal fibroblasts and all tumor cell lines. However, the P_NN_ promoter was not methylated in HpaII sites of normal fibroblasts and tumor cell lines that express *GluR6* transcripts. Such methylation was not observed in cell lines that show no detectable transcript.

Methylation-dependent expression is not a unique phenomenon for GluR6. In fact, a variety of tumor suppressor genes such as *p16*, *p21*, *p14*, *Rb*, *p19*, and *APC* in human cancers are transcriptionally silenced by methylation of CpG dinucleotides [20,21,22,23]. It is noteworthy that transcripts specific for neuronal promoter P_N_ were not expressed in normal fibroblasts and tumor cells. Although glioblastoma cells show expression of the P_N_-specific transcript, the functional status of the encoded protein can only be confirmed after sequencing the transcript in the tumor cells. The P_NN_ promoter, however, regulates the expression of the *GluR6* gene in normal fibroblasts and other cells described here. The effects of aza-cytidine and TSA confirm the transcriptional silencing of P_N_-specific genes in tumor cells due to promoter methylation and/or histone deacetylation. The differential methylation of P_N_ and P_NN_ promoter sequences further confirms the expression of neuronal and non-neuronal *GluR6* variants. It has been well established that methylated CpG dinucleotides interact with methyl-CpG binding proteins and recruit chromatin deacetylases to repress gene transcription [24].

Considering the location of the senescence causing activity on chromosome 6q and the presence of *GluR6* in the minimal sequence responsible for senescence, it is reasonable to predict some role of *GluR6* gene isoforms in this process. Although our data do not provide a mechanistic explanation for the senescence-causing activity of GluR6, other reports in the literature provide some insights about possible mechanisms. Mutations in or deletions of *GluR6* have been characterized in lymphocytic leukemia [13], and glutamate/kainic acid-induced nerve cell death is attributed to GluR6-mediated signaling (5). In fact, such signaling appears to occur by the interaction of PSD95, β-catenin, and synapse-associated proteins (SAPs) with a nerve-specific isoform of GluR6 [4,25,26]. These interactions involve the C-terminus domain of neuronal GluR6 isoforms. The differences in the C-termini of neuronal and non-neuronal GluR6 isoforms suggest that these isoforms manifest their effects via unique intracellular proteins and signaling pathways.

It is pertinent to mention that alterations in metabotropic receptors have also been shown in several human cancers [27,28]. The metabotropic receptors functionally intersect with ionotropic receptors in oligodendrocytes [29], and mutations in metabotropic receptor GRM1 have been observed in melanocytic anemia [30]. The extrapolation of such a relationship between metabotropic and ionotropic receptors suggests roles of GluR6 that warrant their further characterization in human cancers.

In conclusion, our results demonstrate differential expression of *GluR6*/*GRIK2* transcript variants in neuronal and non-neuronal tumor cells. The transcriptional silencing of the *GluR6A* transcript variant in non-neuronal cells is due to selective methylation of the corresponding promoter in these cells. These observations suggest the non-ion channel role of GluR6 variants in epithelial tumor cell lines and set the foundation for investigating tumor-specific roles of non-neuronal GluR6 variants.

## Data Availability

Not applicable.
